# Mutation, drift and selection in single-driver hematologic malignancy: Example of secondary myelodysplastic syndrome following treatment of inherited neutropenia

**DOI:** 10.1371/journal.pcbi.1006664

**Published:** 2019-01-07

**Authors:** Tomasz Wojdyla, Hrishikesh Mehta, Taly Glaubach, Roberto Bertolusso, Marta Iwanaszko, Rosemary Braun, Seth J. Corey, Marek Kimmel

**Affiliations:** 1 Systems Engineering Group, Silesian University of Technology, Gliwice, Poland; 2 Department of Pediatrics, Cleveland Clinic, Cleveland, OH, United States of America; 3 Department of Cancer Biology, Cleveland Clinic, Cleveland, OH, United States of America; 4 Clinical Pediatrics, Division of Hospital Medicine, Stony Brook Children's Hospital, Stony Brook, New York; 5 Department of Statistics, Rice University, Houston, TX, United States of America; 6 Department of Preventive Medicine–Division of Biostatistics, Northwestern University, Chicago, IL United States of America; 7 Department of Engineering Sciences and Applied Mathematics, Northwestern University, Evanston, IL United States of America; 8 Department of Translational Hematology and Oncology Research, Cleveland Clinic, Cleveland, OH, United States of America; 9 Department of Bioengineering, Rice University, Houston, TX, United States of America; University at Buffalo - The State University of New York, UNITED STATES

## Abstract

Cancer development is driven by series of events involving mutations, which may become fixed in a tumor via genetic drift and selection. This process usually includes a limited number of driver (advantageous) mutations and a greater number of passenger (neutral or mildly deleterious) mutations. We focus on a real-world leukemia model evolving on the background of a germline mutation. Severe congenital neutropenia (SCN) evolves to secondary myelodysplastic syndrome (sMDS) and/or secondary acute myeloid leukemia (sAML) in 30–40%. The majority of SCN cases are due to a germline *ELANE* mutation. Acquired mutations in *CSF3R* occur in >70% sMDS/sAML associated with SCN. Hypotheses underlying our model are: an *ELANE* mutation causes SCN; *CSF3R* mutations occur spontaneously at a low rate; in fetal life, hematopoietic stem and progenitor cells expands quickly, resulting in a high probability of several tens to several hundreds of cells with *CSF3R* truncation mutations; therapeutic granulocyte colony-stimulating factor (G-CSF) administration early in life exerts a strong selective pressure, providing mutants with a growth advantage. Applying population genetics theory, we propose a novel two-phase model of disease development from SCN to sMDS. In Phase 1, hematopoietic tissues expand and produce tens to hundreds of stem cells with the *CSF3R* truncation mutation. Phase 2 occurs postnatally through adult stages with bone marrow production of granulocyte precursors and positive selection of mutants due to chronic G-CSF therapy to reverse the severe neutropenia. We predict the existence of the pool of cells with the mutated truncated receptor *before* G-CSF treatment begins. The model does not require increase in mutation rate under G-CSF treatment and agrees with age distribution of sMDS onset and clinical sequencing data.

## Author summary

Cancer develops by multistep acquisition of mutations in a progenitor cell and its daughter cells. Severe congenital neutropenia (SCN) manifests itself through an inability to produce enough granulocytes to prevent infections. SCN commonly results from a germline *ELANE* mutation. Large doses of the blood growth factor granulocyte colony-stimulating factor (G-CSF) rescue granulocyte production. However, SCN frequently transforms to a myeloid malignancy, commonly associated with a somatic mutation in *CSF3R*, the gene encoding the G-CSF Receptor. We built a mathematical model of evolution for *CSF3R* mutation starting with bone marrow expansion at the fetal development stage and continuing with postnatal competition between normal and malignant bone marrow cells. We employ tools of probability theory such as multitype branching processes and Moran models modified to account for expansion of hematopoiesis during human development. With realistic coefficients, we obtain agreement with the age range at which malignancy arises in patients. In addition, our model predicts the existence of a pool of cells with mutated *CSF3R* before G-CSF treatment begins. Our findings may be clinically applied to intervene more effectively and selectively in SCN patients.

## Introduction

Cancer development is driven by series of mutational events, which may become fixed in a hematologic or non-hematologic tumor via genetic drift. This process usually includes a limited number of driver (advantageous) mutations, and a greater number of passenger (neutral or mildly deleterious) mutations. Driver mutations for several hundred different cancers have been identified by sequencing and functional assays. The relationship between driver and passenger mutations has been investigated using mathematical models representing carcinogenesis in terms of a “tug-of war” between the former and the latter [1, 2]. Another related problem is whether carcinogenesis is driven by acquisition of single point mutations or by saltatory changes amounting to major genome rearrangement events [3, 4]. Mathematical modeling of interactions among multiple drivers has been described by Nowak and Durrett and their colleagues [5–7]. These frequently involve branching processes and related mathematical models [8]. Among stochastic models in hematology, an example is [9]. Hematopoiesis provide the best-characterized system for cell fate decision-making in both health and disease [10], as well as connections between stimuli such as inflammation and cancer [11].

Here, we model a disease evolving on the background of a germline mutation. The acquired driver mutation recurs during tissue expansion phase in fetal life and becomes selectively advantageous in early childhood, leading to development of malignancy. As a prominent example of such disease, we model the important hematologic disorder Severe Congenital Neutropenia (SCN), a monogenic inherited disorder, that acquires new mutations and evolves to secondary myelodysplastic syndrome (sMDS) or secondary acute myeloid leukemia (sAML). This model is similar to the “fish” graph of Tomasetti and Vogelstein [12]; however the latter is more comprehensive and involves multiple driver case. Here, we use tools of population genetics and population dynamics to model progression from SCN to sMDS and dissect the contributions of mutation, drift and selection at different stages of an individual’s life. More specifically, we consider:

1. In an individual primed by an inherited genotype, the driver mutation occurs recurrently in the embryonic expansion stage, although these mutations do not necessarily confer selective advantage.
2. At birth, due to environmental and behavioral factors or treatment, the driver mutation acquires a selective advantage in a tissue or organ, while the driver mutation may or may not recur as frequently any more.
3. The mutant driver variant increases in frequency due to selection, and eventually it dominates the stem cells of the tissue or the organ, contributing to development of disease.

Accordingly, SCN is most commonly due to germline mutations in *ELANE*, which encodes the neutrophil elastase [13]. SCN is characterized by the near absence of circulating neutrophils, which renders the child, typically an infant, susceptible to recurrent life-threatening infections. The introduction in the 1990s of recombinant granulocyte colony-stimulating factor (G-CSF) to increase circulating neutrophils, markedly improved survival and quality of life for SCN patients [14].

SCN often transforms into sMDS or sAML [15, 16]. Clinical studies have demonstrated a strong association between exposure to G-CSF and sMDS/AML [17–21]. Mutations in the distal domain of the Granulocyte Colony-Stimulating Factor Receptor (*CSF3R*) have been isolated from almost all SCN patients who developed sMDS/AML [22, 23]. Clonal evolution over approximately 20 years was documented using next generation sequencing and quantification of *CSF3R* allele frequency variation in an SCN patient who developed sMDS/sAML [24]. Strikingly, out of four different mutations in *CSF3R*, one persisted into the leukemic clone but the other three were lost, supporting the assumption of different selective values in the presence of G-CSF that underlies our model. As clonal evolution is a central feature in cancer [25–28] and next generation sequencing has revealed complex genomic landscapes, SCN may provide a simpler real-world example to study cancer development.

Two opposing paradigms have been proposed for cell fate decision making in blood cells: stochastic hematopoiesis (based on variability observed in cultured bone marrow cells as first suggested by McCulloch and Till [29]) and deterministic, or instructive, hematopoiesis (growth factor-driven production of specific blood cell types) [30, 31]. In spite of substantial experimental findings, particularly recent single-cell measurements [32], the two opposing theories await a grand synthesis. Disease-accompanying dynamics have been variously modeled over the years as deterministic or stochastic[10]. SCN may also provide a simpler real-world example to study cell fate determination.

Little is known about the molecular mechanism(s) by which SCN leads to myeloid malignancy and how important are the truncating mutations such as *CSF3R D715* in this process. Notwithstanding the exact molecular mechanism by which the *CSF3R* truncation mutants lead to sMDS/AML, two pivotal questions concerning the population dynamics and population genetics of the mutant clones are: (i) whether the *CSF3R* truncation mutants are present before application of G-CSF, and (ii) whether G-CSF administration increases mutation rate in hematopoietic stem cells with *ELANE* mutation. If the answer to the first question is affirmative, then the presence of a small subpopulation of *CSF3R* truncation mutants among infants with SCN might be of prognostic value and a preventive therapy might be sought. Concerning the second question, determination whether G-CSF is mutagenic or provides a selective pressure may influence the degree G-CSF therapy is conducted, e.g. should it be more or less aggressive.

**Fig 1:**
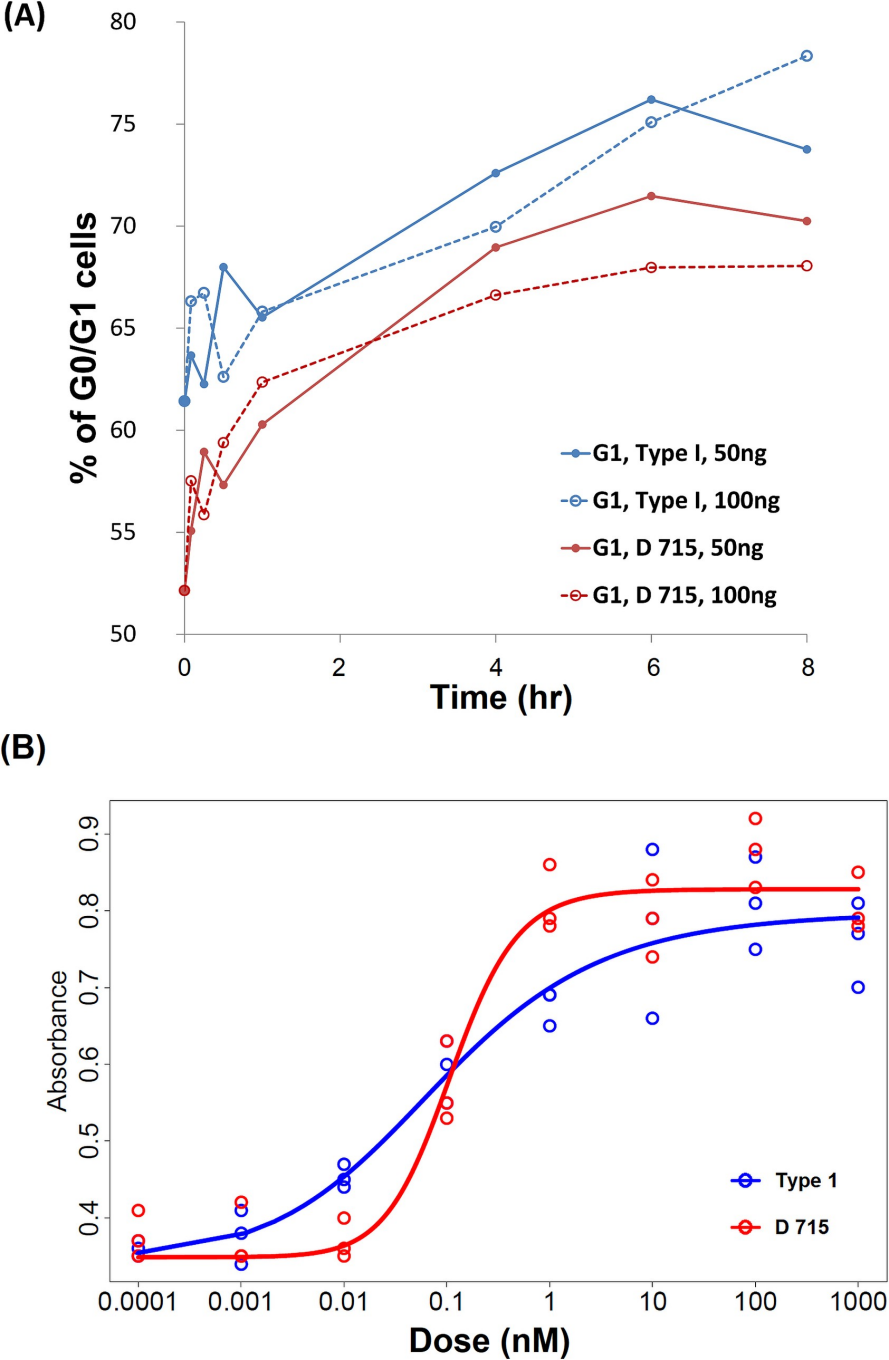
**(A). Effect of CSF3R (wild type or D715) stimulation on cell cycle**. Ba/F3 cells expressing either wild type (Type I, blue) or mutant (D715, red) CSF3R were stimulated with two different doses of G-CSF (50 ng/ml and 100 ng/ml) and were analyzed using flow cytometry to determine the effect of G-CSF dose on receptor subtype activation on cell cycle progression. The cells were treated as detailed in the **Methods**. Flow cytometry data for each time point were analyzed using FlowJo employing the built-in cell cycle module. The data are represented as percentage of G1/G0 cells obtained from the FlowJo analysis plotted against time. Following an initial transient, from the 1 hr time point the G1/G0 percentage differences between cells with the Type I and D715 receptors have been on the average equal to 6.05% for 100 ng of G-CSF and 4.28% for 50 ng of G-CSF, respectively.

To provide insight into the course of this disease and its clinical management, we propose a novel model of the emergence and fixation of *CSF3R* truncating mutations, which also follows the paradigm outlined earlier on. We note that the most common of these mutations associated with transition to sMDS is the *CSF3R D715*. However, at the resolution level of our model, we are not able to make more specific distinctions nor to consider coexistence or competition of more than one truncation mutant. The model assumes that the answer to question (i) is affirmative, but it is negative to question (ii). The model’s hypotheses are:

1. An inherited mutation in *ELANE* causes SCN;
2. *ELANE* mutations coexist with the non-mutated allele in an intracellular environment in which *CSF3R* truncating mutations occur spontaneously at a low rate;
3. During fetal life, the number of stem cells and committed cells expands quickly, which results in high probability of approximately $10^{1}-10^{2}$ *CSF3R* truncation mutant cells; these would be of no consequence had their number not expanded further;
4. Administration of pharmacologic G-CSF early in life exerts selective pressure, providing *CSF3R* mutants with selective advantage. This assumption is supported by experiments, in which growth differential of wild-type and truncation mutant depends on the G-CSF concentration (**Fig 1**).

1. Onset of sMDS is equivalent with replacement of normal hematopoietic bone marrow by mutant hematopoietic stem cells, their progeny, and release of factors that suppress normal hematopoiesis. At the level of resolution of our model, we are not able to make more specific distinctions.

**(B) Effect of CSF3R (wild type vs. D715) on cell proliferation**. Ba/F3 cells expressing either wild type (Type I) or mutant (D715) *CSF3R* were treated with increasing doses of recombinant human G-CSF (ng/ml) and proliferation was measured by the MTT assay performed in triplicates in a 96 well plate. The data are raw absorbance values at 600 nm and represent the three replicates plotted against increasing dose of G-CSF, fitted using least squares by Hill-type curves (Type I, blue, D715 red). For details of experimental procedures see the **S1 Appendix**. Fitting and statistical procedures are explained in the **Methods**.

We show that hypotheses 2 and 3 are needed, by first building a proof-of-principle simple Moran process (a stochastic model used in population genetics) with no expansion, which fits the data only if it is started by a cell population including $⁓10^{1}-10^{2}$ cells expressing a *CSF3R* truncation mutation. Then we show that this number of *CSF3R* truncation mutant cells can be produced in the late fetal period expansion of hematopoiesis in bone marrow. Existence of the pool of *CSF3R* truncation mutant cells before exposure to G-CSF can be discovered only by deep targeted sequencing. Then we follow up with a full-fledged comprehensive model, which accounts for the important detail of time change of the size of the hematopoietic system, but which confirms the conclusions of the proof-of-principle model.

## Methods

### Measuring the selective advantage of D 715 mutant cells

#### Experiment

As shown in **Fig 1A**, the fraction of Ba/F3 cells expressing either wild type (Type I, blue) or mutant (D715, red) CSF3R that reside in the G1/G0 phase has been measured, employing flow cytometry. Technical details are found in the **S1 Appendix**. Briefly, following release from the starvation cell cycle arrest, cells were stimulated with two different doses of G-CSF (50 ng/ml and 100 ng/ml) and were analyzed using flow cytometry to determine the effect of G-CSF dose on receptor subtype activation in terms of cell cycle progression. Flow cytometry data for each time point were analyzed using FlowJo software [33] employing the built-in cell cycle module. The data are represented as percentage of G1/G0 cells obtained from the FlowJo analysis plotted against time. After an initial transient, from the 1 hr time point the G1/G0 percentage differences between cells with the Type I and D715 receptors have been on the average equal to 6.05% for 100 ng of G-CSF and 4.28% for 50 ng of G-CSF, respectively. The data points are derived from flow cytometry, and because it measures thousands of cells, statistical fluctuations play a minor role and therefore the differences are statistically “highly significant”.

G1/G0 cell fraction as a measure of selective advantage of cycling cells. We employ a simple cell cycle model to relate the G1/G0 cell fraction to the cell growth rate and selective advantage. Mathematical foundations can be found for example in ref. [34, 35]. Briefly, suppose that the interdivision time T of cell in a population is constant and that the residence time $T_{1}$ in the G1/G0 is also constant (relaxing these assumptions is possible, as implicit from [34, 35], but it does not affect the first-order approximation we need here). Let us also suppose that the efficiency of divisions α (probability that a progeny cell enters the cell cycle) is constant. Under these hypotheses, the expected cell growth after initial transients becomes exponential with rate $\lambda =\ln\left(2\alpha \right)/T$, i.e. the cell count at time t is equal to

$$N\left(t\right)=N\left(0\right)e^{\lambda t}$$

The fraction φ of cells in G1/G0, tends to the following value

$$\varphi =\frac{1-e^{-\lambda T_{1}}}{1-e^{-\lambda T}}$$

This latter expression can be solved for the ratio $\psi =T_{1}/T$

$$\psi =\frac{\text{ln}\left\{1-\varphi \left[1-{\left(2\alpha \right)}^{-1}\right]\right\}}{-\text{ln}\left(2\alpha \right)}$$

Suppose now that we consider another (“asterisked”) population of cycling cells, which has respective parameters denoted with superscript ${(}^{*}).$Let us also assume that the difference in cell division times between the two populations is due only to shortening of the G1/G0 phase

$$T-T^{*}=T_{1}-T_{1}^{*}=\Delta$$

Let us note that in the experiment described earlier on, we measure the ratios φ (G1/G0 fraction in Type I receptor cells) and $\varphi^{*}$ (G1/G0 fraction in D 715 receptor cells). Following some algebra, we obtain an estimate of Δ in the terms of φ and $\varphi^{*}$ given we assume T and α and $\alpha^{*}$

$$\Delta =\frac{\left(\psi -\psi^{*}\right)}{1-\psi^{*}}T$$

where ψ and $\psi^{*}$ have been already expressed in the terms of φ and $\varphi^{*}$.

Selective coefficient in the Moran model is defined as s=r-1, where r is the ratio of probabilities that the replacent for a withdrawn (divided) self-renewing cell is a mutant (in this case an “asterisked” cell) and, respectively, wild-type. This interpretation is consistent with the confined environment of the bone marrow, in which on the average one progeny cell dies or differentiates, leaving the self-renewing cell pool. This leads to expression

$$s=r-1=\frac{\lambda^{*}-\lambda }{\lambda }$$

**Fig 2:**
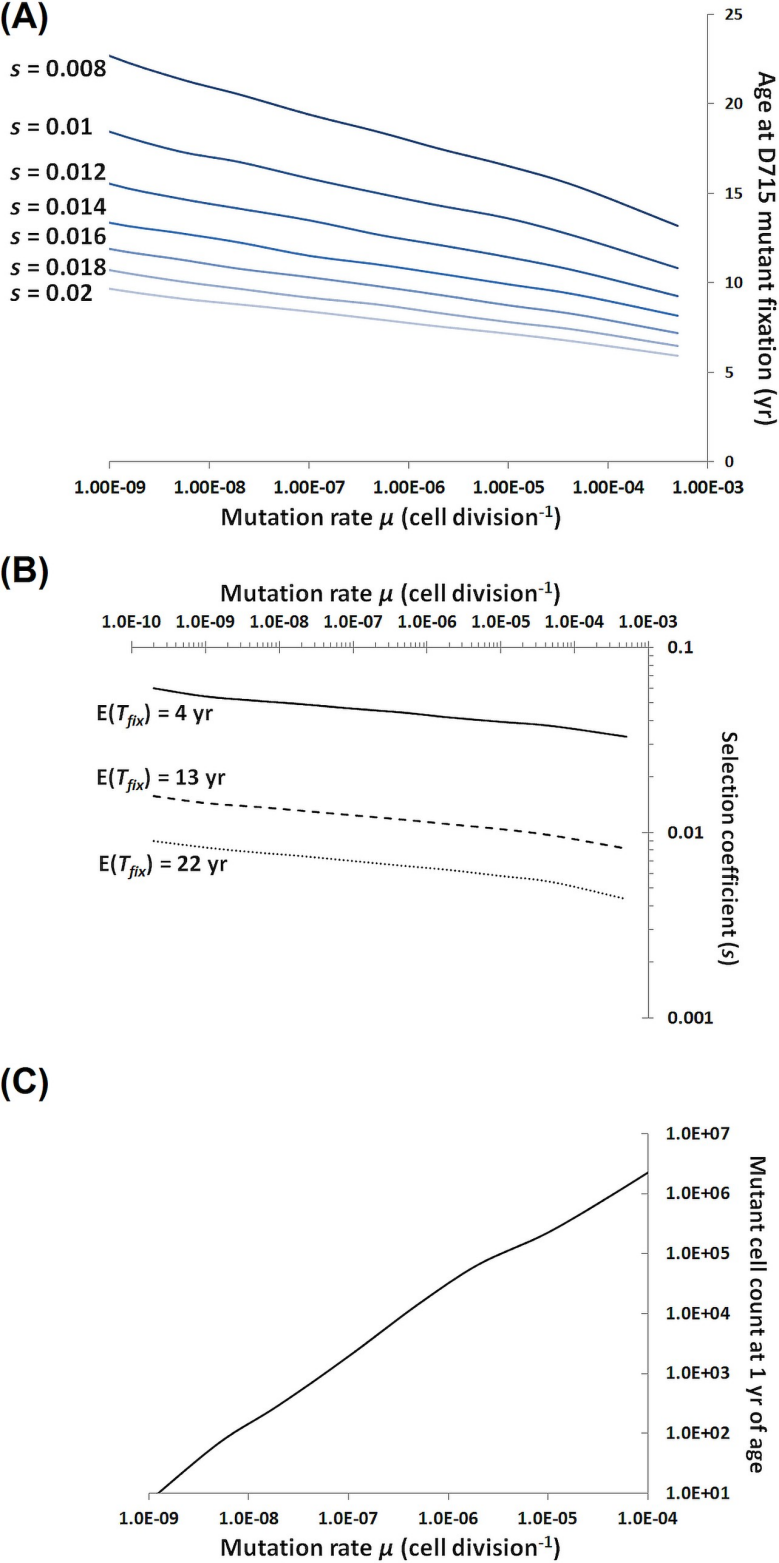
**(A)** Age at which the d715 mutants replace the normal cells in the CMP compartment, as predicted by the modified Moran model with varying cell population size, for a range of mutation rate and selection coefficient values. **(B)** Selection coefficient values corresponding to the ages at replacement of 4 years old (solid line), 13 years old (dashed line), and 22 years old (dotted line) for a range of mutation rates values. **(C)** Mutant cell count at age of 1 year for a range of mutation rate values.

This interpretation allows understanding the bounds on the selection coefficient provided by the experiment. We accepted T=4 days, and we may start from assuming $\alpha =\alpha^{*}=1$ (perfect division efficiency). Suppose that in approximate agreement with the experiment, we consider φ=0.7 and $\varphi^{*}=0.66$, which leads to ψ=0.621 and $\psi^{*}=0.567$, and to Δ=0.503. In turn, this provides $\lambda =0.173{\;d}^{-1}$ and $\lambda^{*}=0.198{\;d}^{-1}$ which leads to s=0.144. Lowering equally the division efficiencies of both cell types changes the selection coefficient only slightly. However, if the “asterisked” (mutant) efficiency is lowered to $\alpha^{*}=0.93$, the selective advantage of faster proliferating cells shrinks to almost 0. Consistently with this, we use the range of s-values from 0.005 to 0.1 (Table 1) and from 0.008 to 0.02 (**Fig 2**), which stay below the upper bound provided by s=0.144.

**Table 1:** Results of the computations of the expected time to fixation.

s	i	μ (cell division^-1^)	$E[T_{0}|T_{N}<T_{0}]$ (yr.)
0.005	240	2.01×10^−08^	90.57
0.01	120	1.00×10^−08^	46.05
0.02	60	4.99×10^−09^	23.41
0.03	40	3.31×10^−09^	15.75
0.04	29	2.47×10^−09^	11.90
0.05	23	1.97×10^−09^	9.57
0.1	11	9.57×10^−10^	4.86

Results of the computations of the expected time to fixation of a *CSF3R* truncation mutation with probability of fixation kept at $P[T_{N}<T_{0}]=0.7$, assuming the Moran process with directional selection. Notation: s, selection coefficient; i, mutant count at birth; μ, mutation rate (cell division^-1^). $E[T_{0}|T_{N}<T_{0}]$ is the age at fixation of the *CSF3R* truncation mutant (yr). For detailed hypotheses and derivations, see the present section.

#### Effect of CSF3R (wild type vs. D715) on cell proliferation

Ba/F3 cells expressing either wild type (Type I) or mutant (D715) *CSF3R* were treated with increasing doses of G-CSF (ng/ml) and proliferation was measured using the MTT assay performed in triplicates in a 96 well plate. Full account of experimental procedures is found in the **S1 Appendix**. The data are raw absorbance values at 600 nm and represent the three replicates plotted against increasing dose of G-CSF.

Concerning recalculation from the pharmacological dose to serum concentration of the G-CSF, ref.[36] is providing relevant information. In this publication, see their **Fig 1**, therapeutic doses from 5–15μg/kg body weight resulted in serum concentrations of the order from 1-100ng/ml, with a maximum for the lowest dose of 5μg/kg reached after 4 hours and equal to over 10ng/ml, and remaining over 1ng/ml for 10 days.

To better understand the data, we performed parametric least-square fitting of the Type 1 and D715 data using Hill-like sigmoid curves, which results in clarified visualization of trends with the two curves crossing approximately at G-CSF concentration of 0.1ng. Hill equation is a prototypical sigmoidal curve widely used in systems biology [37]. It has the form

$$y=y_{0}+a\frac{{\left(\text{ln}x-c\right)}^{n}}{{\left(\text{ln}x-c\right)}^{n}+b^{n}}$$

The best least-square fit to the data (separately for Type I and D715 data). Values of estimated coefficients: Common for Type I and D715, $y_{0}=0.349$; Type 1, *a* = 0.449, *b* = 20.802, *c* = -23.309, *n* = 11.149; D715, *a* = 0.479, *b* = 21.075, *c* = -23.278, *n* = 28.255.

In addition, we carried out rigorous testing using one-sided Wilcoxon two-sample rank test of the difference between the Type 1 and D715 data points separately for concentrations >0.1ng/ml (resulting in highly significant difference at p=0.0034), and for concentrations ≤0.1ng/ml (resulting in borderline significant difference in opposite direction at p=0.089). This testing justifies the assertion of higher growth rate of D715 cell in the range of G-CSF concentrations above 0.1ng/ml.

### Proof-of-principle model

#### Moran process with directional selection

**Fig 3:**
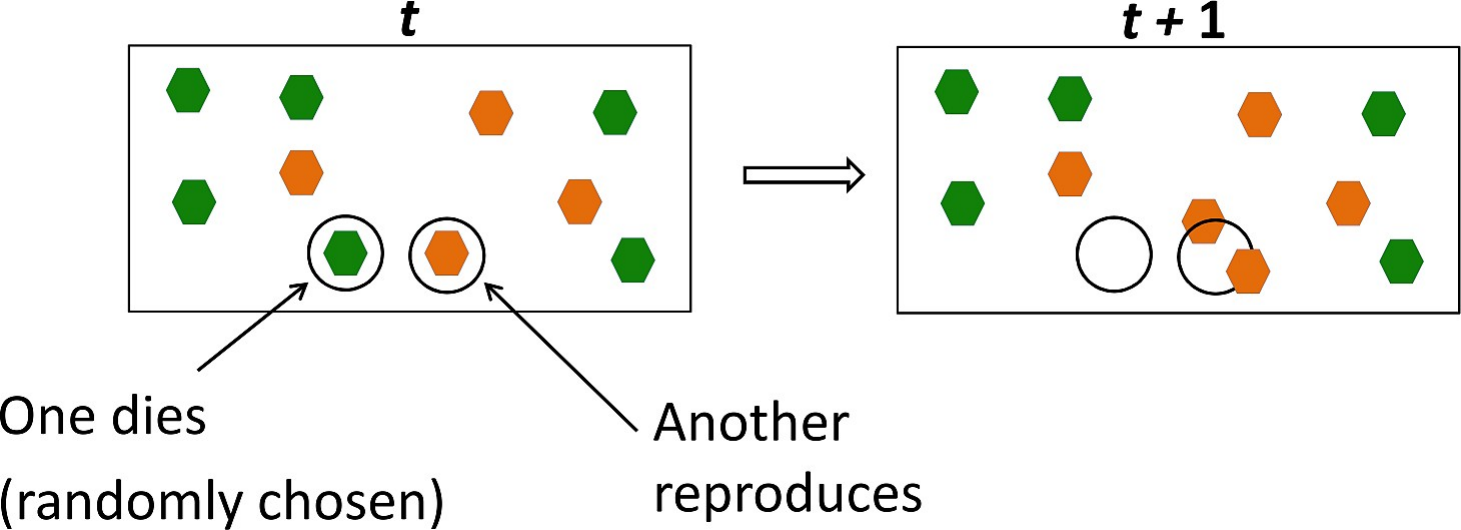
Moran Process with discrete time and directional selection. One of the N cells (i mutants, in orange, and N–i wildtype, in green), present at time t, dies (no preference for type). Its replacement at time t+1 is selected from all cells present at t, with odds (relative fitness) *r* in favor of the mutant. Relative fitness is frequently expressed as r=1+s, where s is called the selection coefficient. For an advantageous mutant, r>1, or s>0.

For a simplified proof-of-principle model of competition between mutant and wild type cells in adult bone marrow, we use the standard Moran process with selection [38]. In this process (**Fig 3**), the population of granulocyte precursors is assumed to be constant, with the proportion of mutants varying in time, and time running in discrete units (such as days or cell-division times). We consider a population of N biological cells, which at time 0 contains i mutant cells. The mutant has a selective advantage defined as the relative fitness r, which is frequently expressed as r=1+s, where s is called the selection coefficient. For an advantageous mutant, r>1, or s>0. Further details are found in the corresponding section of the **S1 Appendix**.

#### Selective advantage in cycling cells

CSF3R is a member of the hematopoietin/cytokine receptor family and functions as a homodimer. The cytoplasmic region consists of a proximal domain essential for proliferation and a distal domain critical for differentiation. Acquired CSF3R mutations have been observed to cluster between nucleotides 2384 to 2522 (residues 715 to 750), resulting in the loss of the distal domain [23]. Epidemiological studies demonstrated that the risk of sMDS/AML increased with the dose of G-CSF [16, 39].

Mutant clones may divide more frequently and/or be less apoptotic. In the simplest case of no cell death, selective advantage can be related to shortening of the interdivision times in mutant cells, relative to normal cells. A proxy for shorter interdivision time is a lower proportion of cells in the G1 phase, as this phase is usually most variable. **Fig 1A** presents a summary of the dynamics of cell cycle distribution of Ba/F3 cells expressing either the full-length wild type CSF3R or the CSF3R D715 mutant following their release from a starvation block. The fraction of cells in G1 in D715 mutants is lower by about 0.05 compared to that in the wild type CSF3R expressing cells, which translates into a growth rate advantage of the CSF3R D715 mutant. The difference is highly statistically significant, as flow cytometry measures thousands of cells per condition.

As an independent check, **Fig 1B** shows a direct comparison of growth curves of Ba/F3 cells expressing either the full-length CSF3R or the truncated CSF3R D715. The dose dependence shows that mutants have a selective advantage over a range of high G-CSF concentrations, whereas for low (normal) concentrations, they are at best neutral or possibly disadvantageous. Relevant laboratory techniques are found in **S1 Appendix**. Similar results were found depending on the particular CSF3R cytoplasmic mutant [40].

#### CSF3R truncation mutations at the fetal-life expansion phase of bone marrow

**Fig 4:**
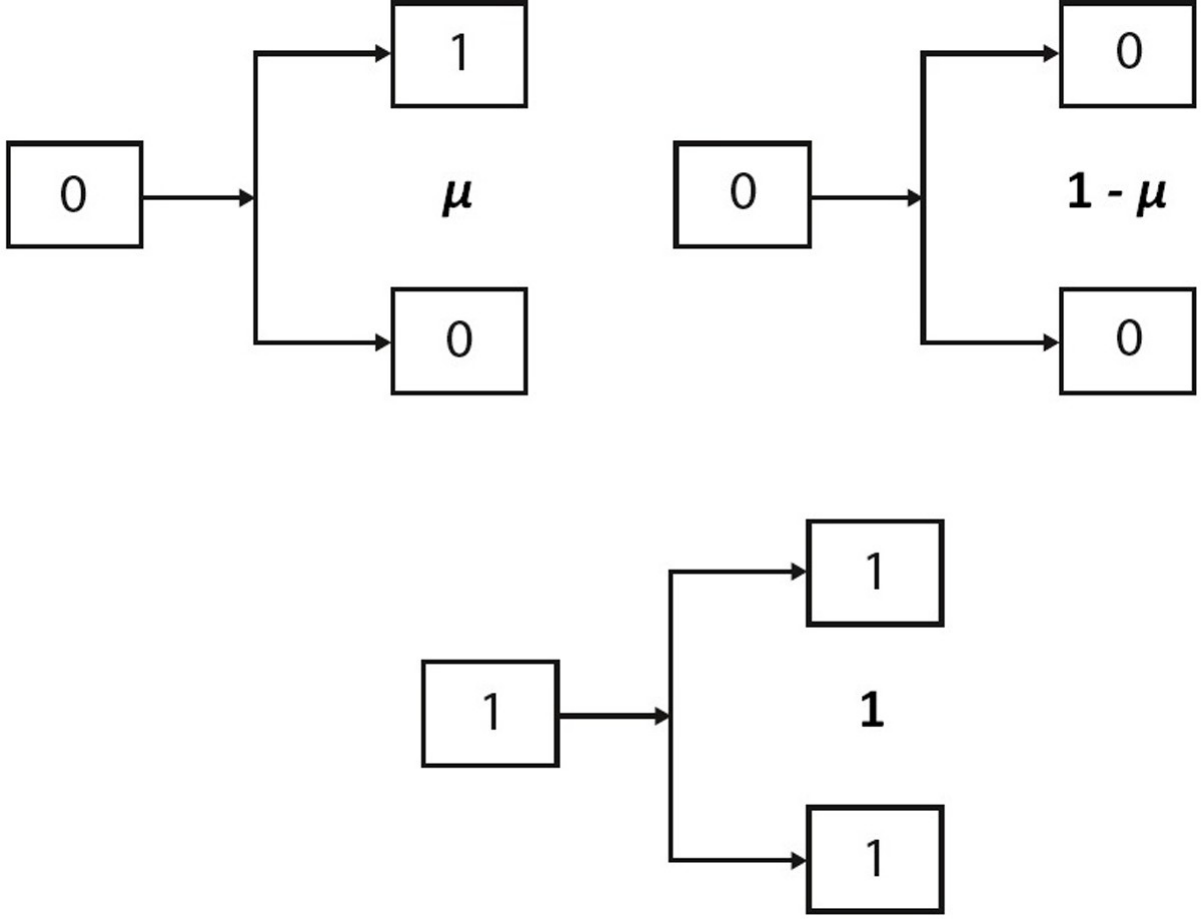
The branching process of cell proliferation with irreversible mutation. Briefly, the cancer cell population is initiated by a single “wild type” (WT) cell, denoted as “0”. At each division, with probability μ, one of the WT progeny cells mutates. Mutants, denoted as “1” produce a pair of mutant progeny. Mutants have the same distribution of the interdivision time as the WT cells.

Hematopoiesis in the human fetus moves from the liver into bone marrow about 90 days before the end of the pregnancy [41, 42]. The requirement of more than one mutant cell present at time *t* = 0 of the Moran process can be satisfied as follows. Suppose that *CSF3R* truncation mutations occur during the embryonic bone marrow expansion stage. In this time interval, because of the rapid expansion on the bone marrow, cell proliferation and mutation can be described using the time-continuous Markov branching process model [8], originally developed by Coldman and Goldie in a different context [43, 44]. The assumptions are as follows (**Fig 4**):

1. The stem cell population (here, pooled Hematopoietic Stem Cells or HSC, and Common Myeloid Progenitors or CMP) is initiated by a small number $N_{0}$ of “wild type” (WT) cell that already acquired the *ELANE* mutation.
2. Interdivision time of WT cells is a random variable from an exponential distribution with parameter λ. Accordingly, mean interdivision time of WT cells is equal to 1/λ.
3. At each division of a WT cell, with probability μ, one of the progeny cells acquires the *CSF3R D715* mutation. *CSF3R* truncation mutants always produce *CSF3R* truncation mutants when dividing.
4. At the expansion phase, mutants are assumed selectively neutral (have the same parameter λ as WT cells).

Mathematical details are found in the corresponding section of the **S1 Appendix**.

### Comprehensive model of fixation of CSF3R truncation mutants

#### Modeling the age-dependent changes of sizes of the bone marrow cell compartments

**Fig 5:**
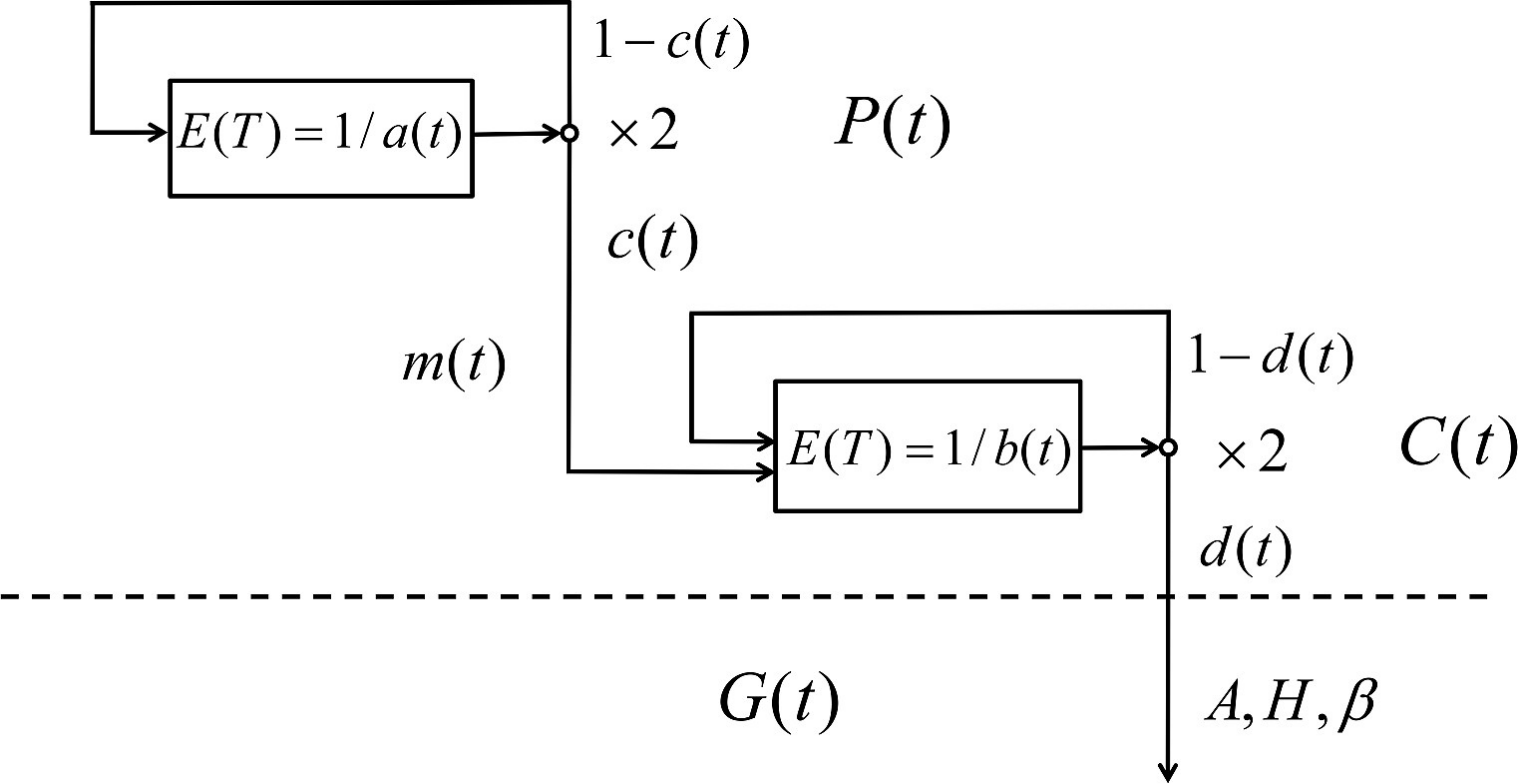
Deterministic model of age-related dynamics of pluripotent and committed cells and granulocytes. P(t), C(t), and G(t) are the numbers of pluripotent cells (HSC compartment), committed cells (CMP compartment), and peripheral granulocytes at time t, respectively; m is the ratio of the committed cells in the hematopoietic cell lineage associated with the granulocyte line (assumed to be m=1/4);aand c are the proliferation rate and self-renewal probability of the P cells; and, similarly, b and d are the proliferation rate and self-renewal probability of the C cells. E(T) denotes expected interdivision time of a cell.

**Fig 6:**
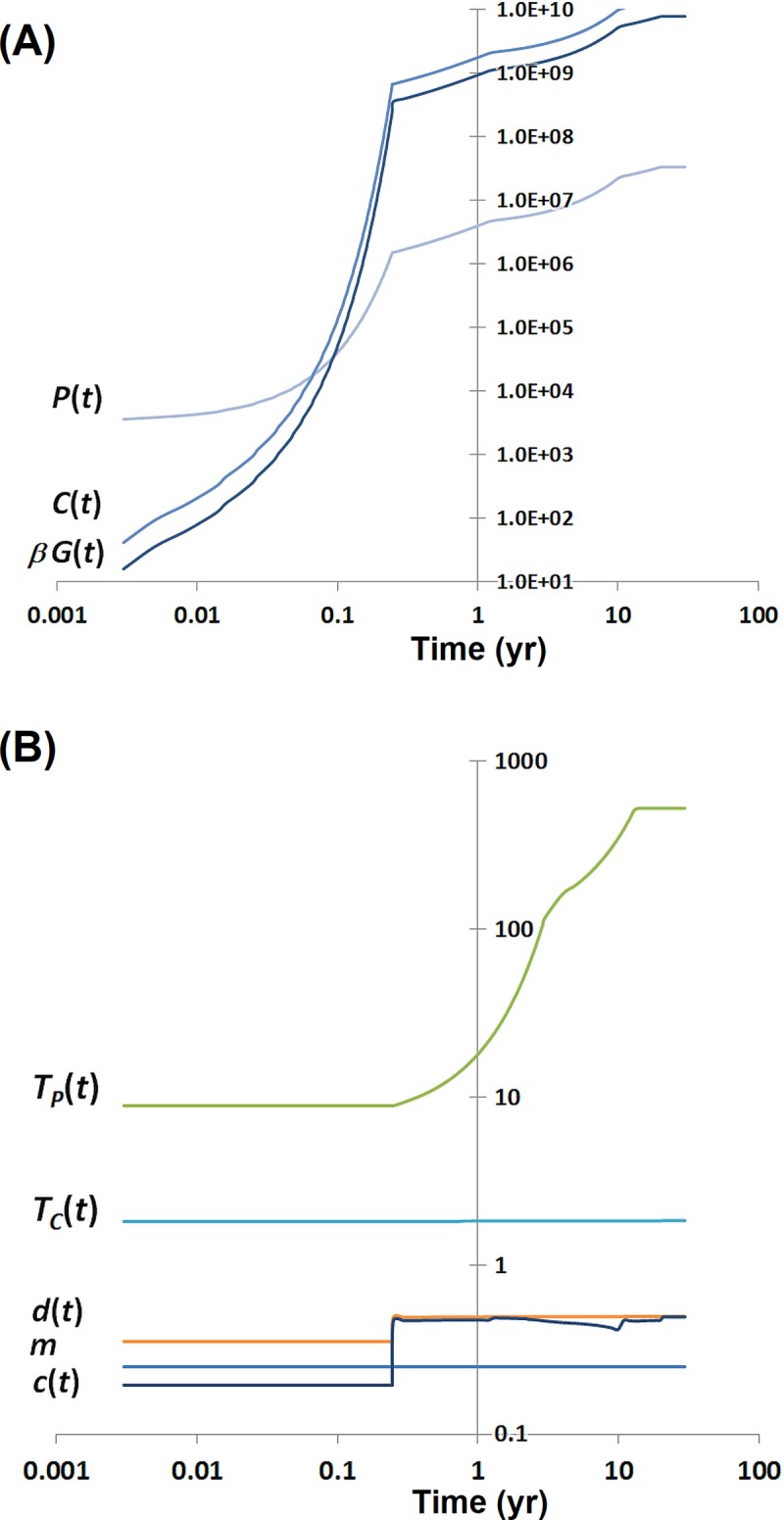
Estimated dynamics of human hematopoiesis in the bone marrow, starting from its onset in the final weeks of fetal life. (For additional details see the twin sections titled *Estimates of the parameters of the model of age-dependent dynamics of the granulocyte arm of the hematopoietic system*, in the Results and in the **S1 Appendix**). (A) Counts of the HSCs (P(t)) and CMPs (C(t)), and the rate of granulocyte production per day (βG(t)), as a function of time (individual’s age), calculated for human body weight following the Theron’s formula. (B) Time (individual’s age) dependent parameters of the mathematical model of the human hematopoietic system: expected time (days) between HSC divisions $T_{P}\left(t\right)=1/a(t)$, expected time (days) between CMP divisions $T_{C}\left(t\right)=1/b(t)$, maturation probability of the HSC c(t), and differentiation probability of the CMP, d(t). Ratio of the committed cells in the hematopoietic cell lineage associated with the granulocyte lineage is assumed equal to m=1/4 for all ages. Please note the log-log scale of the graphs.

For more precise simulations, we build a model of dynamics of the hematopoietic stem cells (HSC) these latter more properly defined as HSC and long term culture-initiating cells (LTC-IC) [45], and of the common myeloid progenitors (CMP), which give rise to neutrophils and/or monocytes. The model schematic is depicted in **Fig 5**. Dynamics of these cell populations are represented in the compartmental model by a system of three differential equations following the model of Arino and Kimmel [46]. The equations of the model are explained in the corresponding section of the **S1 Appendix** and in the legends to **Figs 5**and **6.**

Our model is consistent with the lifetime changes in the bone marrow volume and the HSC interdivision times [47, 48], distinguishes between the periods before and after birth, and accounts for the body mass and HSC proliferation changes during childhood and adulthood. This requires that some of the model parameters change with time t (individual’s age). We found that it is sufficient to vary (i) the mean interdivision time of the HSC equal to $T_{p}=1/a(t)$, (ii) the maturation probability of the HSC equal to *c(t)*, and (iii) the differentiation probability of the CMP equal to *d(t)*. See **S1 Appendix** for explanation of mathematical symbols. The time (age) patterns of these and other coefficients that fit the data in refs. [47, 48], are depicted in **Fig 6B**.

The model accommodates two apparently contradictory observations in the data viz. that (i) the interdivision times of the HSC dramatically increase over the lifetime, and (ii) the granulocyte volume remains proportional to the body weight [47, 48]. Surprisingly, this can be achieved by relatively small changes in the cell maturation and differentiation coefficients. In the real-life system, this is accomplished by nonlinear regulatory feedbacks, with possible configurations similar as in ref. [45]. However, for our purposes, it is sufficient to assume that differentiation coefficients c(t) and d(t) vary with time (**Fig 6B**). Mathematical details and parameter estimates are found in the corresponding sections of the **S1 Appendix**.

#### Model of expansion of the CSF3R truncation mutant in the bone marrow in the form of the Moran process with variable population size, directional selection, and recurrent mutation

In contrast to the standard Moran process, this model assumes that the population size (cell count) *N(t)* varies in time. The population consists of cells of two types: wild type (WT; cells that already acquired the *ELANE* mutation) and *CSF3R* truncation mutants (M). For the WT-cells and M-cells equally, life lengths depend on individual’s age. Upon death of a cell, another randomly chosen cell proliferates. Selective advantage is represented by a bias in choice of proliferating cell (as in the standard Moran model. WT-cell may irreversibly mutate into a M-cell at rate μ. Mathematical details are found in the corresponding section of the **S1 Appendix**.

Technically, the model is limited to the CMP compartment of the bone marrow, since (1) cells in this compartment respond to G-CSF signaling as opposed to the HSC, which most likely do not, and (2) CMP compartment is much larger than the HSC one. Further stages of granulocyte precursors are assumed to only transmit the descendants of the mutated cells into peripheral blood and tissue and not to have even limited self-renewal properties. Before the GCS-F treatment is initiated, the mutants do not have selective advantage (i.e. s=0). Advantage appears at the time G-CSF treatment is administered, assumed to be six months after birth.

## Results

### Proof-of-principle model

#### Mutation rate, selection and age at sMDS onset in the proof-of-principle model

In this section, we consider a simple Moran model with the mutants being cells carrying the *CSF3R* truncation mutation. Since around 70% of sMDS associated with SCN carry this mutation, this means that fixation of the mutant (i.e., elimination of the wild-type *CSF3R* in the SCN population) occurs with probability 0.7 (ref. [23]). Assuming this and a given selection coefficient of the mutant over the wild type, we calculate (see **S1 Appendix**) the expected time to fixation and the required number of mutant cells at time 0 of the model (corresponding to birth of the individual). **Table 1**presents results of the computations of the expected time to fixation of a *CSF3R* truncation mutation with probability of fixation kept at P=P[fixation]=0.7, assuming the Moran process with directional selection. The parameter values are consistent with the adolescent phase values in the accurate model of bone marrow expansion, as well as with the experiment-based estimates of the selective advantage of cells harboring the *CSF3R* truncation mutation, as outlined further on.

#### Determination of the expected time to fixation of the CSF3R truncation mutant and the required count of mutant-harboring cells at the end of fetal bone marrow expansion

Expected times to fixation of the *CSF3R* truncation mutant were computed by first solving Eq. (1) in the **S1 Appendix** to find the initial count i of mutants required for P=0.7, given the summary number of HSC and CMP cells N = 1.98×10^8^ cells/kg ×75 kg (the average adult body weight) and selection coefficient s varying in a wide range.

In mathematical terms, if the probability of fixation of the mutant is provided by the Eq. (1) in the **S1 Appendix**

$$P\left[T_{N}<T_{0}\right]=\frac{1-{\left(1-s\right)}^{i}}{1-{\left(1-s\right)}^{N}},$$

and for large N and small i, the expected time to fixation (given that fixation occurs), is asymptotically equivalent to [38]

$$E\left[T_{N}|T_{0}>T_{N}\right]=\frac{2\text{ln}N-\text{ln}i}{s},$$

then we can solve the first of these two equations for i, assuming $P\left[{T_{N}<T}_{0}\right]=0.7$ to obtain

$$i={\text{log}}_{1-s}\left\{1-0.7\left[1-{\left(1-s\right)}^{N}\right]\right\}$$

and then substitute this into the second equation to obtain

$$E\left[T_{N}|T_{0}>T_{N}\right]=\left\{2\text{ln}N-\text{lnln}\left\{1-0.7\left[1-{\left(1-s\right)}^{N}\right]\right\}-\text{lnln}\left(1-s\right)\right\}/s$$

The latter has to be divided by the number of cell divisions per year (assumed to be equal to 90 as in Stiehl et al. [45]) to obtain time in years.

The resulting mutant cell counts and the expected age at fixation of the *CSF3R* truncation mutation are collected in the second and fourth column of **Table 1**, respectively.

For s from the interval (0.02, 0.1), the estimates of the expected time to fixation of the mutant (time when only mutant allele remains) belong to the interval [4.86, 23.41] (yr), and are approximately consistent with the timing of the sMDS onset. From the European SCN Registry data, age at diagnosis of SCN with sMDS and *CSF3R* mutation is 13 ± 9 years. However, it is necessary that at the time G-CSF treatment begins, i = 11–60 mutant cells are already present in the cell population (second column of **Table 1**).

#### Determination of the mutation rates required to obtain the mutant cell counts at birth

To shed light at the possibility of this number of mutants being present approximately at the birth time, we solved Equation (3) in the **S1 Appendix** to obtain the estimates of mutation rate μ which makes it possible, absent selection by G-CSF, to obtain the corresponding initial mutant count i(t)=i in the range 11–60 in the fetal hematopoietic population of HSC and CMP expanding to the size of about $N\left(t\right)=N$ = 1.98×10^8^ cells/kg ×5 kg (the approximate infant body weight).

Mathematically, under the branching process model (**S1 Appendix**), we obtain the equations for the expected (average) number N(t) of normal and $i\left(t\right)$of mutant cells [8]

$$N\left(t\right)=N_{0}\text{exp}[\left(1-\mu \right)\lambda t],i\left(t\right)=N_{0}\{\exp\left(\lambda t\right)-\text{exp}[\left(1-\mu \right)\lambda t]\}$$

Eliminating time from the relationship between N(t) and $i\left(t\right)$, we obtain that the expected number of mutant cells is equal to

$$i\left(t\right)=N\left(t\right)-N_{0}^{\mu }N{\left(t\right)}^{1-\mu }\;≅\;N\left(t\right)-N{\left(t\right)}^{1-\mu }\;≅\;\mu N\left(t\right)\text{ln}\;N\left(t\right)$$

where N(t) (resp. $N_{0}$) is the number of cells after (resp. before) expansion. The approximation on the right-hand side is valid for moderate $N_{0}$ and small μ. Inverting this expression, we obtain the mutation rates for a given expected number of mutant cells.

The resulting mutation rates per cell division, ranging from 9.57×10^−10^ to 4.99×10^−09^, with cell cycle time of the CMP (which dominate in the pooled HSC and CMP population; see further on) assumed equal to 4 days (as in Stiehl et al. [45]), do not exceed the values considered normal for human cells (**Table 1**).

As for **Table 2**, we select the mutation rates and expected times to fixation to be a subset of those in **Table 1**and compare the respective selection coefficients stemming from the simplified model and the comprehensive model. A more complete review of possible parameter combination is possible using **Fig 2**as a nomogram; see further on.

**Table 2:** Comparison of estimates of selection coefficients needed for fixation of the *CSF3R mutant*.

Age at *CSF3R* truncation fixation (yr)	Mutation rate μ (cell division^-1^)	Selection coefficient (s), based on proof-of-principle model	Selection coefficient (s), based on comprehensive model
4	1 × 10^−9^	0.10	0.054
13	2.5 × 10^−9^	0.04	0.014
22	5 × 10^−9^	0.02	0.008

Comparison of estimates of selection coefficients (rounded) needed for fixation of the *CSF3R* truncation mutant at a given age, based on the proof-of-principle and comprehensive models, with the corresponding mutation rates (rounded) corresponding to simulations underlying estimates in **Table 1**.

### Comprehensive model of fixation of CSF3R truncation mutants

The results of the proof-of-principle modeling summarized in **Table 1**suggest that it is feasible to build a more comprehensive model consistent with normal hematopoiesis as well as mutation and selection mechanisms modified by the age-dependent cellularity of the bone marrow and administration of pharmacological G-CSF.

#### Estimates of the parameters of the model of age-dependent dynamics of the granulocyte arm of the hematopoietic system

We assume that the total number of HSC, CMP, and granulocytes is proportional to body weight, which increases according to Theron's formula [49] from 3.4 kg at the birth time to 75 kg in the adult life. The exact numbers of these cells at time t are calculated based on the estimates given by Stiehl et al. [45] (in that paper’s Online Supplement, Scenario 2). Further details are available in the corresponding section of the **S1 Appendix**. **Fig 6**presents the age-trajectories of model coefficients as well as those of the cell numbers in the two compartments and the flux rate of mature granulocytes into blood.

#### Mutation rate, selection and age at sMDS onset in the detailed model including recurrent mutation

The main results of the paper are obtained by application of the comprehensive model of expansion of the *CSF3R* truncation mutants in the bone marrow. As described in Methods section, the model hypotheses are consistent with the age-dependence of the cell number in the CMP compartment. We disregard the influx of mutants arising in the HSC compartment, since this is a relatively minor influence compared to their number in the CMP, the increase of which is driven by their selective advantage following the G-CSF treatment. Because the Moran model with recurrent mutation has mutant fixation probability equal to 1, it applies strictly speaking only to the 70% of cases with *CSF3R* truncation mutation fixed at the sMDS diagnosis.

**Fig 2A** depicts the age at which the *CSF3R* truncation mutants replace the normal cells in the CMP compartment. The age at replacement reaches the mean value of 13 years observed in clinical data for a range of parameter values, including mutation rate expected for human genome (ca. 10^−9^) and modest selection coefficient values such as 0.014 consistent with small selective advantage expected based on **Fig 3**though not explicitly quantifiable. **Fig 2B** depicts the values of the selection coefficient s, which for a given mutation rate lead to sMDS onset at 4, 13 and 22 years, respectively.

Comparison of simulations in **Fig 2**(comprehensive model) and computations in **Table 1**(simplified model), leads to the conclusions summarized in **Table 2**. Estimates of the selection coefficients needed for fixation of the *CSF3R* truncation mutant at ages 4, 13 and 22 years obtained from the comprehensive model are about 2–3 times higher than those based on the simplified model, with the corresponding mutation rates adjusted to those required in the simplified model. This difference stems from two facts: (1) the simplified model does not account for the change of the hematopoietic system performance with age, and (2) the comprehensive model includes recurrent mutations of *CSF3R* over the lifetime not only in the fetal period. In both models, the mutation rates and selection coefficients required seem to be within acceptable ranges.

Another graph, in **Fig 2C**, depicts the corresponding mutant count at 1 year of age. We see that at mutation rates ranging over 10^−9^–10^−7^, the mutant count at birth remains in the 10^1^–10^3^ range. These results confirm the proof-of-concept analysis and show that in the range of “normal” human mutation rates, the number of mutants at birth is of the order of 10^2^—practically undetectable even by very deep sequencing.

## Discussion

Here we presented a model of fixation of a *CSF3R* truncation mutant in the transition from an inherited neutropenia to sMDS: from the expansion phase in the prenatal hematopoietic tissues, to initiation of the G-CSF treatment, to expansion of the mutant, and to replacement of the normal bone marrow by the pre-leukemic mutants. By modifying the simple Moran model of population genetics, we provided an explanation for the evolution of sMDS in about 70% of cases in which *CSF3R* truncation mutant acts as an oncogenic driver. We first used a proof-of-concept two-stage model including the initial creation of the mutant clone before the selective agent G-CSF has been applied, followed by the period of selective pressure after initiation of treatment. We followed up with a more comprehensive model, which used the estimates of age-dependent productivity changes in hematopoietic stem cells, obtained based on telomere shortening estimates by the Abkowitz and Aviv groups [47, 48].

Our model provides a real-world setting that may further illuminate principles of clonal hematopoiesis of indeterminate potential, first described as age-related clonal hematopoiesis. As recently summarized by [50], HSC clonality and association with malignancy begins with somatic genetic lesions in adult stem cells that accumulate and persist and that “given a large enough population (of HSC), every base pair in the genome will be mutated within at least one HSC”. Further, “these mutations provide the substrate for clonal selection”. The original and distinctive feature of our present model is to show that mutations occurring during the bone marrow expansion in the fetal period are likely to play a major role in creating this substrate.

The expected times to fixation of the *CSF3R* truncation mutant (4–22 years) are consistent with the timing of the sMDS onset. According to data published from the European SCN Registry data, the average age at diagnosis of SCN with sMDS and *CSF3R* mutation is 13 ± 9 years [51]. The 70% fixation probability requires 11–60 “initial” cells harboring the mutation. We experimentally validated our mathematical model by measuring the growth advantage of the CSF3R D715-expressing cells and found a significant growth advantage (**Fig 1A**). Further validation will require next generation sequencing of specimens from these rare patients. Qiu et al. [52] recently reported that this truncation mutation also permits granulocytic precursors to avoid apoptosis.

Our comprehensive model is based on the hypothesis that the rate of cell division after birth, when the rapid expansion of bone marrow slows down, is still very high. Hence, acquisition of new mutants during that phase is still substantial. However, selection is the force that leads the mutant-receptor cells to dominate. This also means that supply of new mutants in the expansion phase might not be necessary for the disease to emerge. However, it is likely that proliferation slows down by one or two orders of magnitude, depending on exact characteristics of subtypes of stem cells. Then in order to fit the data, somewhat higher selection coefficients are needed. In that case, the comprehensive model will behave approximately as the “proof of the concept” model, i.e. most of the mutant are supplied in the marrow expansion stage.

An alternative hypothesis states that an inherited neutropenia induces a maladaptive increase in replicative stress and higher mutation rate in HSC that contributes to transformation to sMDS/AML [53]. However, measurements of the mutation burden in individual hematopoietic stem/progenitor cells (HSPCs) from SCN patients failed to support that. CD34^+^CD38^-^ cells were sorted from blood or bone marrow samples and cultured for 3–4 weeks on irradiated stromal feeder cells. The exomes of the expanded HSPC clones were sequenced with unsorted hematopoietic cells from the same patient served as a normal control. The average number of somatic mutations per exome was 3.6 ± 1.2 for SCN, compared to 3.9 ± 0.4 for the healthy controls. Those patient-derived findings support our model. Our conclusions require that the mutation rate per site per cell division equals about 10^−9^, which is consistent with normal mutation rate in human genome.

This latter issue warrants discussion since the somatic mutation rate in humans is about two orders of magnitude higher than the germline mutation rate, as suggested by [54]. However, a recent paper by Milholland et al. [55], argues that this former (somatic rate) is of the order of 10^−9^ per base per mitosis, while the former (germline rate) is of the order of 10^−11^ per base per mitosis (**Fig 1B** in that paper). Moreover, as seen in our **Fig 2**, using the 10^−7^ mutation rate [54] would only slightly change our conclusions.

Two other mechanisms drive the expansion of the CSF3R truncation mutants, (i) the initial CSF3R truncation mutant cell clones arising in the expansion phase of fetal hematopoietic bone marrow and (ii) competitive advantage of the CSF3R truncation mutant harboring cells at later ages, hypothetically due to increased G-CSF pressure. “Mutator phenotype” does not need to be invoked in the SCN progression to sMDS.

A characteristic feature of human cancers is their wide heterogeneity with respect to extent of involvement, genotype, and rate of progression and spread [56]. This variability contrasts markedly to induced animal tumors, which grow at a relatively uniform rate. sMDS/AML secondary to SCN is not an exception, with onset varying from 1 to 38 years of age. Previously, we constructed a stochastic model of the SNC→sMDS→sAML transition based on stochastic events [9]. It considered each new mutation to provide more selective advantage to the arising clone. This linear structure of mutation conferred desirable simplicity to modeling but was not necessarily realistic. In the framework of multitype branching processes and special processes such as Griffiths and Pakes branching infinite allele model [57, 58], more complicated scenarios might be contemplated. Interestingly, the model of ref. [9] suggests that the spread in the age of onset of sAML is not due solely to stochastic nature of clone transitions, but requires a large variability in proliferative potential from one affected individual to another.

Similar effect can be predicted in the Moran process. According to [38], the time course of the Moran process under mutant selective advantage can be split into three periods: (1) relatively long period from small number of mutant cells to a threshold, followed by (2) a much shorter period from the threshold to near-fixation of the mutant, and (3) a relatively long period to complete fixation. Accordingly, once the mutant count exceeds certain threshold, the process accelerates. This results in the spread of times to fixation depending at least as strongly on the selection coefficient as on the “intrinsic” randomness. This justifies the approach we took in this study, to concentrate on the effects of the selection coefficient. In addition, determination of the threshold may help establish a target for monitoring the progress of the disease. Gaining more insight will require a further study.

While our model advances the understanding of multistep progression to cancer with a real-world condition and application to the clinic, other factors could be incorporated. These include: a correlation between G-CSF dosage for neutrophil recovery in SCN patients and the risk of malignant transformation and acquisition of an additional mutation, such as *RUNX1*, in the evolution to sAML.

In the current report, we focus on a single aspect of the SCN-related leukemogenesis: expansion of *CSF3R* truncation mutant cells leading to the sMDS transformation. The model we present here provides potentially testable hypotheses (i) the *CSF3R* truncation mutants are present in $10^{1}-10^{2}$ cells before G-CSF treatment is applied and (ii) a slight selective advantage of the *CSF3R* truncation mutant-harboring cells under G-CSF pressure is sufficient to lead to their expansion. The second hypothesis seems to be consistent with findings in ref [53]. Current dogma holds that clonal dynamics in relation to the development of sMDS/AML are highly heterogeneous and unpredictable. Our model supports the clinical value of more accurate disease surveillance with next generation sequencing and better timing of therapeutic interventions, such as stem cell transplantation.

## Supporting information

S1 Appendix Supporting methods. (DOCX)
