# Supplementary material for: Mutation, drift and selection in single-driver hematologic malignancy: Example of secondary myelodysplastic syndrome following treatment of inherited neutropenia
Source: PLoS Comput Biol. 2019 Jan 7;15(1):e1006664. doi: 10.1371/journal.pcbi.1006664 (PMC6336352; doi:10.1371/journal.pcbi.1006664)
Supplement: S1 Appendix — (DOCX) [file pcbi.1006664.s001.docx]

**S1 APPENDIX**

**Population Genetics Model of Secondary Myelodysplastic Syndrome:**

**From Mutated Receptor to Age Distribution at Diagnosis**

Appendix includes mathematical details of the models introduced in an intuitive way in the Models and Data section and detailed description of the experimental methods applied in the cell proliferation study (results presented in **Figure 1**).

**Mathematical details**

***Moran process with directional selection***

As described in Models and Data, we consider a population of $N$ biological cells, which at time 0 contains $i$ mutant cells and $N-i$ wild type (WT) cells. The mutant has a selective advantage expressed by the relative fitness $1+s$, equal to the ratio of average progeny count of the mutant to that of the WT. For an advantageous mutant, the selection coefficient $s>0$. Mathematically, the Moran process is a time-continuous state-dependent random walk with two absorbing boundaries at $0$ and $N,$ with transition intensities

$$q_{i,i+1}=\frac{\left( N-i \right)i}{N}, q_{i,i-1}=\frac{i\left( 1-s \right)\left( N-i \right)}{N}, i=0,1,\ldots,N$$

$$q_{i,i}=-\left( q_{i,i+1}+q_{i,i-1} \right)$$

and equal to 0 otherwise.

Let us denote by $T_{0}$ and $T_{N}$ the times to extinction or fixation of the mutant. Under Moran process, the probability of fixation of the mutant is asymptotically equal to [38] (Durrett 2008)

$P\left[ {T_{N}<T}_{0} \right]=\frac{1-{(1-s)}^{i}}{1-{(1-s)}^{N}}$ (1)

while for large $N$ and small $i$, the expected time to fixation (given that fixation occurs), is asymptotically equivalent to [38]

$E\left[ T_{N} | T_{0}>T_{N} \right]=(2\ln N-\ln i)/s$ (2)

***Recurrent mutations at the fetal-life expansion phase of bone marrow***

As explained in Models and Data, the assumptions of the theory are as follows (**Fig. 3**):

1. The stem cell population (here, pooled Hematopoietic Stem Cells or HSC, and Common Myeloid Progenitors or CMP) is initiated by a small number $N_{0}$of “wild type” (WT) cell that already acquired the *ELANE* mutation.

2. Interdivision time of WT cells is a random variable from an exponential distribution with parameter $\lambda$. Accordingly, mean interdivision time of WT cells is equal to $1/\lambda$.

3. At each division of a WT cell, with probability $\mu$, one of the progeny cells acquires the *CSF3R D715* mutation. *CSF3R D715* mutants always produce *CSF3R D715* mutants when dividing.

4. At the expansion phase, mutants are assumed selectively neutral (have the same parameter $\lambda$ as WT cells).

Under these assumptions, we obtain the equations for the expected (average) number $N(t)$ of normal and $i\left( t \right)$of mutant cells [8] (Kimmel and Axelrod 2015)

$N\left( t \right)=N_{0}exp[(1-\mu)\lambda t$], $i\left( t \right)=N_{0}\{\exp\left( \lambda t \right)-exp[(1-\mu)\lambda t$]}

Eliminating time from the relationship between $N(t)$ and $i\left( t \right)$, we obtain that the expected number of mutants is equal to

$i(t)=N(t)-N_{0}^{\mu}{N\left( t \right)}^{1-\mu}\cong N(t)-{N\left( t \right)}^{1-\mu}\cong\mu N\left( t \right)\ln N(t)$ (3)

where $N(t)$ (resp. $N_{0}$) is the number of cells after (resp. before) expansion. The approximation on the right-hand side is valid for moderate $N_{0}$ and small $\mu$.

***Modeling the age-dependent changes of sizes of the bone marrow cell compartments***

As stated in the corresponding section of Models and Data, **Fig. 4** presents the compartmental model in the form of a system of three differential equations following the model of Arino and Kimmel [46] (Arino and Kimmel 1986). The equations of the model have the form

$$\dot{P}\left( t \right)=a\left( t \right)\left[ 1-2c\left( t \right) \right]P\left( t \right)$$

$\dot{C}\left( t \right)=2a\left( t \right)m\left( t \right)c\left( t \right)P\left( t \right)+b\left( t \right)\left[ 1-2d\left( t \right) \right]C\left( t \right)$ (4) $\dot{G}\left( t \right)=2Ab\left( t-H \right)d\left( t-H \right)C\left( t-H \right)-\beta G(t)$

where $P(t)$, $C(t)$, and $G(t)$ are the numbers of HSC, common myeloid progenitors (CMP), and peripheral granulocytes respectively at time $t$; $m$ is the ratio of the committed cells in the hematopoietic cell lineage associated with the granulocyte line (assumed to equal $m = 1/4$); $a$ and $c$ are the proliferation rate and self-renewal probability of the $P$ cells; and, similarly, $b$ and $d$ are the proliferation rate and self-renewal probability of the $C$ cells. The third equation of the system (4) implies that with relatively low rate of change of parameters of the system (scale of months and years), the number of peripheral granulocytes $G(t)$ is with high accuracy following the number $C(t)$ of $C$ cells multiplied by the time-dependent factor $Ab(t)d(t)/\beta$, since the delay $H$ (on the order of days) is negligibly small compared to the lifetime scale and the time derivative $\dot{G}\left( t \right)$ is very small considering the rates of changes in the system. Further, estimation of the proportionality constant $A$ is not necessary, since the number of granulocytes and turnover rate are known (see Results).

***Model of expansion of the CSF3R D715 mutant in the bone marrow in the form of the Moran process with variable population size, directional selection, and recurrent mutation***

As stated in the corresponding section of Models and Data, in contrast to the standard Moran process, this model assumes that the population size (cell count) $N(t)$ varies in time, with time $t$ chosen as continuous or discrete (continuous in our case). The population consists of cells of two types: wild type (WT) and mutant. For the WT and mutant cells equally, life lengths are independent with distributions characterized by time-dependent hazard rates $\lambda(t)$*.* We note that the tail probability that the cell born at time $t$ dies after time $t+s$ is equal to $exp[-\int_{t}^{t+s} \lambda\left( u \right)du]$ (Cox and Oakes 1984). Upon death of a cell, another randomly chosen cell proliferates; for mathematical simplicity, it is usually assumed that it may be, among other, the deceased cell that proliferates; this standard assumption does not alter the overall behavior of the process. Directional selection is modeled by a bias in choice of the cell that proliferates. A WT-cell is chosen with weight $1-s$, where $s\in(0,1)$ is the selection coefficient. In the model with recurrent mutation, a WT-cell may irreversibly mutate with hazard rate $\mu$ and become a mutant.

Although modeling of the Moran process is relatively straightforward, even if recurrent mutation and varying population size are considered, one technical challenge for simulations is the immense count of cells in the bone marrow, which translates into long simulation times. To alleviate this, we employed a -leaping algorithm (Gillespie, Hellander et al. 2013), which can be used even when rare events, such as point mutations of cells from small cell groups (either wild type or mutants), may occur in the model. Time in *-*leaping scheme defines the duration of single simulation step. In short, in each step of the simulation we mark all events in the model as either non-critical (affecting only major cell types in population) or critical. Then, we determine the waiting time *W* to the first critical event and we accept this event only if the waiting time is shorter than . In this case, the waiting time *W* becomes a single simulation step time for the current step. At the end of the single step process we execute all non-critical events that occurred during this step. In our Moran model, we assume that critical events are all events (deaths, births or changes from wild type to mutant cells) affecting cells from a group (either, wild type or mutant cells) with size lower than 10. The number of events of each type from the non-critical group is sampled from a Poisson distribution. Waiting time to the next critical event $W(t)$ is a random variable distributed exponentially with parameter $T(t) / S,$ where $T(t)$ is the cell interdivision time at time $t$ and $S$ is the sum of rates of all critical events. The parameter of Poisson distribution used to calculate the number of simulated events of a particular type is given by $\min(W(t),\tau)x(\tau)/T$, where *τ* is the rate of events of this type.

***Estimates of the parameters of the model of age-dependent dynamics of the granulocyte arm of the hematopoietic system***

We assume that the total number of HSC, CMP and granulocytes is proportional to body weight, which increases according to Theron's formula (So, Farrington et al. 2009) from 3.4 kg at the birth time to 75 kg in the adult life. The exact numbers of these cells at time $t$ are calculated based on the estimates given by [45] (Stiehl, Ho et al. 2014) (in that paper’s Online Supplement, Scenario 2), which are 4.47×10^5^ HSC cells and 1.97×10^8^ CMP cells per 1kg of body weight. We assume that the turnover rate of granulocytes ($\beta G(t)$ in the model) is equal to 1.7×10^9^ cells per day per 1 kg of body weight (Basu, Hodgson et al. 2002, Lahoz-Beneytez, Elemans et al. 2016). Finally, we assume that the HSC interdivision times change during the lifetime according to the results obtained by Aviv [48] (Sidorov, Kimura et al. 2009), based on computations involving telomere shortening. Age-specific times between HSC divisions (denoted $1/a(t)$ in the model) are as follows, using our interpolation:

- in the last 90 days of fetal life (the period when hematopoiesis relocates to the bone marrow), it equals 9 days;
- during first 3 years after birth, it changes exponentially from 9 to 146 days;
- between 3 and 13 years of age, it changes exponentially from 146 to 521 days; and
- in adult life, it equals 521 days.

The exponential pattern of growth has been assumed as an approximation of an unknown increasing convex pattern. To calculate the values of the remaining parameters over the lifetime, we numerically solve the first two differential equations using the assumptions listed above. Then, at each time step $dt$ of the numerical calculations (equal to 10^-4^ day, or ~9 seconds, in our calculations), we fit the values of the unknown parameters in the way that matches the changes in the known number of cells.

As a result, we obtain that

- expected interdivision time of the CMP, $1/b(t)$, changes only slightly during the lifetime, around the value corresponding to the 1.85 d average time between cell divisions,

- maturation probability of the HSC, $c(t)$, oscillates from 0.473 at birth, to 0.494 at 15 m of age, 0.42 at 10 yr. of age, and to 0.5 in the adult life,

- differentiation probability of the CMP, $d(t)$, increases monotonously from 0.495 at birth to 0.5 in the adult life.

**Figure 5** presents the age-trajectories of model coefficients as well as those of the cell numbers in the two compartments and the flux rate of mature granulocytes into blood.

**Supplemental Methods (regarding material in Fig. 1)**

***Cell Culture*** Ba/F3 cell expressing either wt CSF3R (GRI) or the d715 CSF3R (d715) cells were grown in 1X RPMI (Cellgro) containing 10% fetal bovine serum (FBS, Atlanta Biologicals), 1% PenStrep (Invitrogen), 1X GlutaMAX (Invitrogen) and 2 ng/ml mIL3 (PeproTech) at 37⁰ C, 5% CO_2_. Cell density was maintained using above conditions between 2 x 10^5^ cells/ml and 1 x 10^6^ cells/ml. For treatment with G-CSF, mIL3 was replaced with appropriate concentration of G-CSF (Filgrastim, Amgen). Serum and IL3 starvation of cells was performed using 1X RPMI media containing 1% bovine serum albumin (BSA), 1% PenStrep and 1X GlutaMAX.

***MTT Cell Proliferation Assay*** Ba/F3 GRI and d715 growing in IL3 containing media at log phase of growth were washed twice with PBS to remove IL3. The cells were then cultured in a 96 well plate, under conditions of increasing dose of G-CSF from 0 to 1000 ng/ml, at a density of 2 x 10^4^ cells/0.1ml/well. The cells were incubated at 37⁰ C, 5% CO_2_ for 48 hours. MTT reagent (3-(4, 5-dimethylthiazolyl-2)-2, 5-diphenyltetrazolium bromide) was added to cells as per the manufacturer protocol (ATCC). Incubation was carried out for 4 hours before adding detergent as per manufacturer’s protocol. After overnight incubation at room temperature (RT), absorbance was measured using a 96 well plate reader (Fluostar Optima) at 600 nm.

***Cell Cycle Analysis by Flow Cytometry*** Ba/F3 GRI and d715 cells growing in IL3 were washed twice with 1X RPMI and starved for 8 hours in 1X RPMI, 1% BSA at 37⁰ C, 5% CO_2_, to achieve cell cycle arrest. After 8 hours of serum and IL3 starvation, cells were released from cell cycle arrest by adding serum (10% final) and G-CSF (50ng/ml or 100ng/ml final) and incubated at 37⁰ C, 5% CO_2_ for 0, 7.5, 15, 30, 60, 240 and 480 min. Post incubation cells were fixed immediately in ice cold 0.5% paraformaldehyde in PBS. Incubation was carried out for 15 min at RT. Cells were then washed with PEB (1X PBS, 2 mM EDTA, 1% BSA) and permeabilized with ice-cold 70% methanol for 1h. Cells were then washed with PEB and then treated with 100 µg/ml RNase at 37⁰ C, 5% CO_2_. Cells were washed with PEB and then stained with 25 µg/ml propidium iodide in PEB for 20 min at RT in dark. Stained cells were immediately run on a flow cytometer (BD LSRFortessa) and data analyzed using FlowJo software (https://www.flowjo.com/solutions/flowjo).

**Supplemental References**

Basu, S., et al. (2002). "Evaluation of role of G-CSF in the production, survival, and release of neutrophils from bone marrow into circulation." Blood **100**(3): 854-861.

Cox, D. R. and D. Oakes (1984). Analysis of Survival Data, Chapman and Hall.

Gillespie, D. T., et al. (2013). "Perspective: Stochastic algorithms for chemical kinetics." Journal of Chemical Physics **138**(17).

Lahoz-Beneytez, J., et al. (2016). "Human neutrophil kinetics: modeling of stable isotope labeling data supports short blood neutrophil half-lives." Blood **127**(26): 3431-3438.

So, T. Y., et al. (2009). "Evaluation of the accuracy of different methods used to estimate weights in the pediatric population." Pediatrics **123**(6): e1045-1051.
